# Deficiency of RAMP1 Attenuates Antigen-Induced Airway Hyperresponsiveness in Mice

**DOI:** 10.1371/journal.pone.0102356

**Published:** 2014-07-10

**Authors:** Manyu Li, Sarah E. Wetzel-Strong, Xiaoyang Hua, Stephen L. Tilley, Erin Oswald, Matthew F. Krummel, Kathleen M. Caron

**Affiliations:** 1 Departments of Cell Biology and Physiology, University of North Carolina at Chapel Hill, Chapel Hill, North Carolina, United States of America; 2 Department of Genetics, University of North Carolina at Chapel Hill, Chapel Hill, North Carolina, United States of America; 3 Department of Medicine, Division of Pulmonary and Critical Care Medicine, University of North Carolina at Chapel Hill, Chapel Hill, North Carolina, United States of America; 4 Department of Pathology, University of California San Francisco, San Francisco, California, United States of America; Helmholtz Zentrum München/Ludwig-Maximilians-University Munich, Germany

## Abstract

Asthma is a chronic inflammatory disease affecting the lung, characterized by breathing difficulty during an attack following exposure to an environmental trigger. Calcitonin gene-related peptide (CGRP) is a neuropeptide that may have a pathological role in asthma. The CGRP receptor is comprised of two components, which include the G-protein coupled receptor, calcitonin receptor-like receptor (CLR), and receptor activity-modifying protein 1 (RAMP1). RAMPs, including RAMP1, mediate ligand specificity in addition to aiding in the localization of receptors to the cell surface. Since there has been some controversy regarding the effect of CGRP on asthma, we sought to determine the effect of CGRP signaling ablation in an animal model of asthma. Using gene-targeting techniques, we generated mice deficient for RAMP1 by excising exon 3. After determining that these mice are viable and overtly normal, we sensitized the animals to ovalbumin prior to assessing airway resistance and inflammation after methacholine challenge. We found that mice lacking RAMP1 had reduced airway resistance and inflammation compared to wildtype animals. Additionally, we found that a 50% reduction of CLR, the G-protein receptor component of the CGRP receptor, also ameliorated airway resistance and inflammation in this model of allergic asthma. Interestingly, the loss of CLR from the smooth muscle cells did not alter the airway resistance, indicating that CGRP does not act directly on the smooth muscle cells to drive airway hyperresponsiveness. Together, these data indicate that signaling through RAMP1 and CLR plays a role in mediating asthma pathology. Since RAMP1 and CLR interact to form a receptor for CGRP, our data indicate that aberrant CGRP signaling, perhaps on lung endothelial and inflammatory cells, contributes to asthma pathophysiology. Finally, since RAMP-receptor interfaces are pharmacologically tractable, it may be possible to develop compounds targeting the RAMP1/CLR interface to assist in the treatment of asthma.

## Introduction

Asthma is a debilitating chronic disease affecting about 25 million people in the United States [1] that costs taxpayers over $56 billion per year in medical costs and lost productivity [2]. Individuals prone to asthma have chronic inflammation of the airways possibly caused by pre-sensitization of the immune system to substances that are normally innocuous [3]. When these immune cells become activated after recognition of a trigger, smooth muscle contraction, edema, and mucus hypersecretion are triggered, resulting in the appearance of symptoms. In addition to environmental triggers, it is believed that genetic factors pre-dispose individuals to asthma. To date, several GWAS studies have identified SNPs that are correlated with asthma, including loci harboring the IgE receptor [4], [5], cytokines [4], [6], and DNA repair elements [4], highlighting the multifaceted nature of this disease.

Prior to the discovery of the receptor activity-modifying proteins (RAMPs), the exact receptors through which peptides such as calcitonin gene-related peptide (CGRP) and adrenomedullin (AM) acted remained unclear. Although it was believed that these peptides signaled via G-protein coupled receptors (GPCRs), the exact identity of these receptors was highly debated. With the discovery of the RAMPs in 1998, it became clear that this was the mechanism by which ligand specificity and receptor transportation were dictated for some GPCRs [7]. Specifically, McLatchie et al determined that CGRP interacted with the calcitonin receptor-like receptor (*Calcrl* = gene; CLR = protein) in the presence of RAMP1, while the association of RAMP2 with CLR produced an AM receptor [7]. Since this initial study, it has been determined that the interaction between CLR and RAMP3 also produces an AM receptor [8], although this does not appear to be the major AM receptor since *Ramp3^−/−^* mice are viable [9], in contrast to the embryonic lethality exhibited by *Calcrl^−/−^* [10], *Ramp2^−/−^* [11] and *Adm^−/−^* [12] mice. The encoding of each RAMP by individual genes located on separate chromosomes in humans allows RAMP expression and thus, receptor function, to be finely controlled in individual cell populations. Since the RAMPs confer ligand specificity to GPCRs that may recognize multiple ligands, this RAMP-receptor interface has great potential as a reliable pharmaceutical target to allow for specific blockage or activation of a single ligand without altering the signaling of other ligands that utilize the same GPCR. In fact, compounds that block the RAMP1/CLR interface are currently undergoing clinical trials for the treatment of migraine pain mediated by CGRP signaling [13].

Calcitonin gene-related protein (CGRP) is a neuropeptide with widespread expression throughout the central and peripheral nervous systems [14]. This initial study by Rosenfeld et al determined that the lung contains CGRP-positive nerve fibers which tended to localize with the smooth muscle cells of the blood vessels [14], indicating a possible role for CGRP in maintenance of vascular tone. In fact, studies have demonstrated that CGRP is an extremely potent vasodilator [15], which suggests that CGRP may be protective in the context of asthma. However, reports indicating that CGRP may promote inflammation through enhancement of cytokine production [16] seem to suggest the opposite effect of CGRP in the context of asthma. Studies interrogating the role of CGRP during allergic asthma have been conflicting [17], [18], thereby necessitating further studies regarding the role of the CGRP signaling pathway in allergic asthma. Since CGRP signals via the *Ramp1*/*Calcrl* receptor interface, we chose to examine the effect of *Ramp1* loss on airway resistance and inflammation in order to interrogate the CGRP signaling cascade without altering the adrenomedullin signaling pathway. In this study, we found that loss of *Ramp1* attenuated the airway resistance in mice sensitized and challenged with ovalbumin (OVA) compared to similarly treated wildtype animals. These results identify a role for *Ramp1*-mediated signaling in the hyperresponsiveness of the airways in this model of allergic asthma.

## Methods

### Generation of Mice with Targeted Deletion of the RAMP1 Gene

To generate the targeting vectors, a 129S6/SvEv genomic library was screened for phage clones containing the 3′ portion of the *Ramp1* gene using DNA fragments isolated from *hRAMP1* expression plasmids (kindly provided by Dr. Steven Foord, GlaxoSmithKline). Using convenient restriction sites within the genomic clones, the 6.1 kb 5′ region of homology was subcloned into the multiple cloning site of the AMC1 gene-targeting vector that contained a phosphoglycerate kinase-neomycin cassette preceded by a LoxP site and a herpes simplex virus-thymidine kinase cassette. A 1.2 kb PCR amplification fragment containing exon 3 of the *Ramp1* gene and a second LoxP site were inserted between the 5′ region of homology and the neomycin cassette and a second 1.2 kb PCR amplification fragment from the 3′ region of homology was inserted after the neomycin cassette. The final targeting vectors were linearized with Not I before electroporation into TC1 embryonic stem cells from 129S6/SvEvTAC mice following standard gene targeting methods. After applying positive (G418) and negative (gancyclovir) selection, positive embryonic stem cell clones were identified by Southern blot and/or PCR. Then, a CMV-CRE plasmid was transfected into the positive clones and the deletion of exon 3 and the neomycin cassette was confirmed by Southern blot. The ES cells were injected into C57BL6 blastocytes and male chimeric mice that transmitted the targeted allele were bred to 129S6/SvEv females to establish isogenic lines. For PCR-based genotyping of the *Ramp1*-targeted locus the following primers were used: primer 1, 5′-TCATGGGGACCTTTAGGTAAGC-3′ and primer 2, 5′ ACAGCAATCCTTCTACCTCAACAC-3′. This PCR reaction generates a 1.6 kb band for the wildtype allele and a 0.4 kb band for the *Ramp1* knockout allele.

In addition to the *Ramp1^−/−^* mice generated for this study, the previously described *Calcrl^+/−^* [10], *Calcrl^fl/fl^* [11], and SM22-Cre [19] lines were used for these studies. Unless otherwise noted, experimental animals were 4–8 months old and maintained on an isogenic 129S6/SvEv background. Control animals for all experiments consisted of wild type age- and sex-matched littermates. All experiments were approved by the Institutional Animal Care and Use Committee of The University of North Carolina-Chapel Hill and the Institutional Animal Care and Use Committee of the University of California San Francisco.

### Gene Expression Analysis

RNA was isolated from adult tissues using TRIzol reagent (Invitrogen) in accordance with the manufacturer’s protocol. To prevent genomic DNA contamination, RNA was treated with DNaseI prior to cDNA synthesis with M-MLV reverse transcriptase (Invitrogen, 28025-013). *Ramp1* gene expression was assessed by quantitative reverse transcription (qRT)-PCR using the Mx3000P Real-Time PCR system from Stratagene. Primers for *Ramp1* amplification were 5′-CCTCTGCTTACCTCTGAGATTG-3′ and 5′-ATCTGTGCAGTCTTCCTTGGAGT-3′. The probe sequence for *Ramp1* detection was 5′-FAM-ACACTTCATCACCACTGTGGGCATTCTG-TAMRA-3′.

### Blood pressure measurements and heart weight to body weight measurement

Systolic, mean arterial pressure (MAP), and pulse pressure were determined by the tail cuff method as described previously [20], [21]. In order to determine the heart weight to body weight ratio, body weights were recorded prior to euthanizing the animal. Then, the entire heart was dissected from the chest and the great vessels and any associated connective tissue were removed prior to measuring the heart weight.

### Induction of allergic inflammation and *measurement of airway resistance* (*R_AW_*)

In order to sensitize mice to ovalbumin and generate a model of asthma, an intraperitoneal injection consisting of 20 µg of chicken ovalbumin (OVA, grade V, Sigma-Aldrich) emulsified in 200 µl of aluminum hydroxide adjuvant (Alhydrogel, Accurate Chemical & Scientific) was administered to each animal. Fourteen days later, mice were challenged with either aerosolized saline (PBS) or 1% ovalbumin (OVA) in 30-minute sessions for 5 consecutive days using a whole-body exposure chamber. Twenty-four hours after the last exposure, airway resistance (R_AW_) was measured in anesthetized mice as previously described [22]. Basal R_AW_ measurements were made every 10 seconds for 1 minute prior to serially challenging mice with aerosolized methacholine (MCh) at the following concentrations: 10 mg/mL, 20 mg/mL, and 40 mg/mL. Mice were administered each concentration of MCh for 20 second prior to recording the R_AW_ at 10 second intervals for 2 minutes immediately following each challenge period.

### Whole lung lavage in anesthetized mice

After R_AW_ measurement, the chest was opened, and lungs were promptly lavaged with PBS as described previously [23]. Lavage fluid was kept on ice prior to centrifugation at 360×*g* for 10 min at 4°C. The supernatants were subsequently frozen at –80°C for cytokine analysis.

### Lung histopathology

Immediately following whole lung lavage, the lungs were inflated with 10% neutral-buffered formalin at a constant pressure of 20 cm using a syringe. Upon completion of lung inflation, the trachea was tied off to prevent leakage of fixative. Then the lungs were removed en bloc and immersed in 10% formalin for fixation. Following fixation, the tissues were dehydrated with ethanol in preparation for paraffin embedding. Using the left lobe of the lungs, 5- to 6-µm serial sections were cut and stained with H&E to assess general morphology.

### Cytokine Measurements

The concentration of IL-4 in cell-free bronchoalveolar lavage (BAL) fluid was measured using an ELISA kit (M4000B, R&D Systems, Minneapolis, MN) according to the manufacturer’s protocol.

### Whole lung tissue digestion

Mice were euthanized with an intraperitoneal injection of 2.5% Avertin, followed by removal of the lungs from the chest cavity. The lungs were then placed in 5 ml of RPMI media containing 1.3 MandlU of Liberase and 0.2 mg DNase. The tissue was dissociated using a lung-optimized program on a Miltenyi Biotec gentleMACS machine followed by a 30-minute incubation at 37°C. Using the gentleMACS dissociator, the tissue was homogenized. Red blood cells were lysed, and 2 million cells were plated for antibody staining.

### Antibody staining for RAMP1 and CLR

Lung tissue homogenates were stained with the following conjugated antibodies: anti-mouse CD45 AlexaFluor700 (1∶300; eBioscience), anti-mouse Siglec-F AlexaFluor647 (1∶300; BD Bioscience), anti-mouse Ly6g PeCy7 (1∶400; BioLegend), anti-mouse/human CD11b BV605 (1∶2500; BioLegend), anti-mouse CD11c BV510 (1∶2000; BioLegend). A Zombie NIR fixable viability kit (Biolegend) was used to differentiate live cells from dead cells. To stain for CLR and RAMP1, rabbit anti-CLR (H42; Santa Cruz) and rabbit anti-RAMP1 (FL-148; Santa Cruz) primary antibodies were used, followed by a donkey anti-rabbit AlexaFluor647 (1∶200; Abcam) secondary. The samples were analyzed by flow cytometry on a BD LSRFortessa.

### Statistics

Statistical analyses were performed with student’s t-test. Error bars represent standard error of the means. Differences were considered significant with a *p* value of <0.05.

## Results

### Generation of *Ramp1* knockout mice

**Figure 1:**
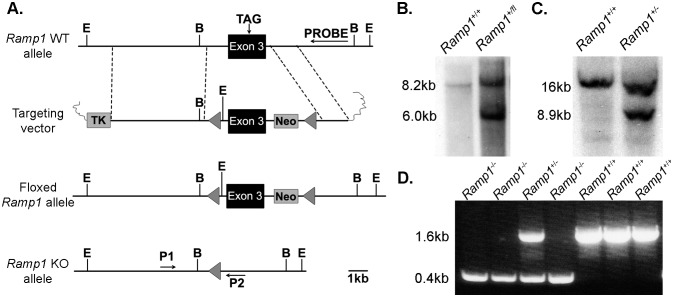
Targeting vector for the generation of *Ramp1* knockout mice. **A.** Targeting vector design for excision of exon 3 from the mouse *Ramp1* gene. (E = EcoRI, B = BamHI) **B.** Southern blot to confirm generation of *Ramp1* floxed allele. **C.** Southern blot confirming the successful generation of *Ramp1^+/−^* after Cre-mediated excision of exon 3. **D.** Routine genotyping using primers 1 (P1) and 2 (P2) shown in panel 1A.

Previous studies have indicated an important role for CGRP in the pathology of asthma [17], [24]. Since CGRP can signal via the *Ramp1/Calcrl* receptor, we were interested in determining whether a loss *Ramp1* could also result in an attenuation of airway hyperresponsiveness after sensitization to ovalbumin and a subsequent challenge period to reflect the findings of Aoki-Nagase et al. Therefore, we developed a global *Ramp1* knockout mouse model, referred to as *Ramp1^−/−^* mice for the duration of this article. Studies performed by other groups have demonstrated that the second and third extracellular α–helices of *Ramp1* contain residues critical for CGRP binding and subsequent cAMP generation [25]. Therefore, we decided to excise exon 3 of isoform 1 of the mouse *Ramp1* gene, which codes for these extracellular loops, to remove these critical residues and prevent the formation of a functional CGRP receptor. Although mouse *Ramp1* has three isoforms, including an isoform that utilizes an alternate exon 3 from the form encoded by isoform 1, three-dimensional structure prediction models of isoforms 2 and 3 indicate that these variants lack the third α–helix and have an incomplete second α–helix. Additionally, isoforms 2 and 3 lack the critical residues described by Simms et al as necessary for CGRP interaction and cAMP generation [25], indicating that it is unlikely that these isoforms can interact with CLR to form a functional CGRP receptor. Using standard molecular biology techniques, we flanked the third exon of the mouse *Ramp1* gene with loxP sites to generate the targeting vector (**Figure 1A**). We confirmed the successful generation of this floxed allele by Southern blotting (**Figure 1B**) prior to transfecting the positive ES cells with a plasmid expressing Cre recombinase driven by the CMV promoter in order to excise exon 3 in all cells, thereby generating *Ramp1^−/−^* embryonic stem cells. We then, confirmed the generation of the *Ramp1* knockout allele by Southern blotting (**Figure 1C**) before using these cells to generate the *Ramp1* ^−/−^ line. Finally, we used the primers shown in Figure 1A to perform routine genotyping of the *Ramp1^+/−^* offspring (**Figure 1D**).

### *Ramp1^−/−^* mice are phenotypically normal

**Figure 2:**
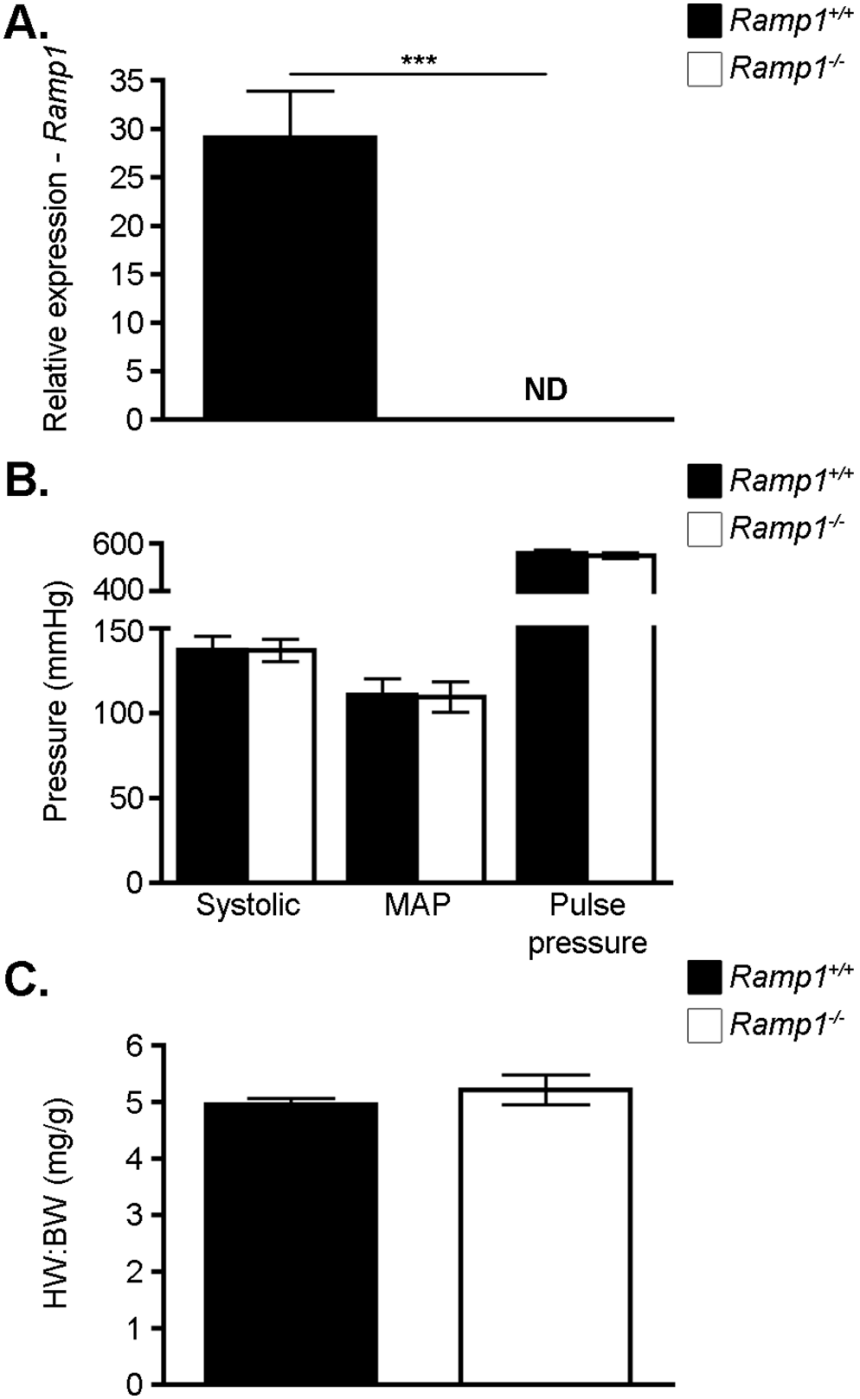
*Ramp1* knockout animals have normal cardiovascular parameters. **A.** Knockdown of *Ramp1* was confirmed by assessing the relative expression level of *Ramp1* in the brain by quantitative RT-PCR. ND = not detected (n = 3 per group) **B.** Mean arterial pressure (MAP), systolic pressure, and pulse pressure were measured by tail cuff. (n = 4 per group) **C.** Heart weight to body weight ratios (HW:BW) ratios were assessed at 2–3 months of age. (n = 4–8) Data represent means±SEM. ***p<0.001.

To ensure that *Ramp1^−/−^* animals do not have basal phenotypes that might interfere with data interpretation, we began our studies by performing a brief phenotypic analysis. We found that *Ramp1^−/−^* animals breed normally, are viable, and have normal lifespans. Our analysis of *Ramp1* expression levels by qRT-PCR using brain tissue revealed that *Ramp1^−/−^* animals do not have any detectable levels of *Ramp1* (**Figure 2A**), confirming the efficient knockdown of *Ramp1* with this targeting strategy. Tsujikawa et al used a different targeting strategy to generate *Ramp1* knockout animals and found that *Ramp1^−/−^* mice were hypertensive [26]. Therefore, we measured the mean arterial pressure, systolic pressure, and pulse pressure in wildtype and *Ramp1^−/−^* animals by tail cuff (**Figure 2B**). Our analysis revealed that *Ramp1^−/−^* animals have blood pressures that are indistinguishable from wildtype controls. We also assessed the heart weight to body weight (HW:BW) ratios of wildtype and *Ramp1^−/−^* mice as a crude readout of potential differences in hypertrophy. We found that the HW:BW ratios of wildtype and *Ramp1^−/−^* mice were identical (**Figure 2C**), supporting the conclusion that *Ramp1^−/−^* hearts are normal. Finally, we found that compared to wildtype animals, *Ramp1^−/−^* mice express approximately 6 times more *Calcrl* in the lung (WT = 210±136 vs. *Ramp1^−/−^*1390±459), indicating a potential for enhanced AM signaling via RAMP2/CLR and RAMP3/CLR complexes.

### Loss of *Ramp1* reduces airway resistance after ovalbumin challenge

**Figure 3:**
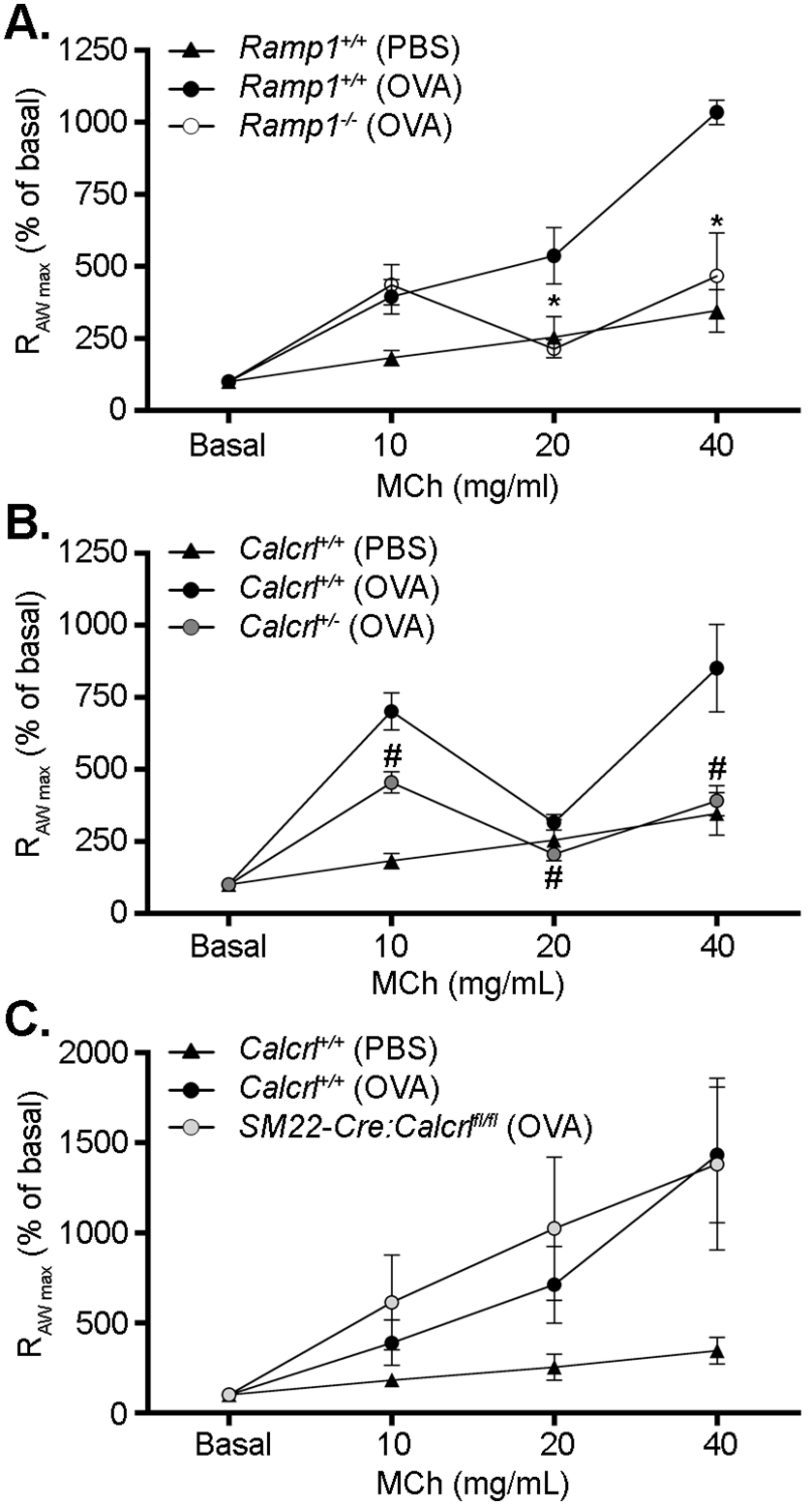
Attenuated airway resistance in sensitized *Ramp1^−/−^* and *Calcrl^+/−^* animals compared to wildtype. **A.** *Ramp1^+/+^* and *Ramp1^−/−^* animals were sensitized to ovalbumin (OVA) as described in the methods section prior to challenging the animals with either aerosolized saline (PBS) or ovalbumin (OVA) for five days. The maximum airway resistance (R_AW max_) at the basal state and after challenge with aerosolized methacholine (MCh) was determined. (n = 3–4 per group) **B.** *Calcrl^+/+^* and *Calcrl^+/−^­* animals were sensitized to ovalbumin prior to challenge as described in panel A. R_AW max_ was determined for the basal state and in response to MCh challenge 24 hours after challenge with aerosolized PBS or OVA. (n = 3–4 per group) **C.** *Calcrl* was specifically knocked down in smooth muscle cells using the SM22-Cre mouse. These *SM22-Cre:Calcrl^fl/fl^* mice and wildtype controls were then sensitized and challenged as described in panels 3A and 3B. (n = 4 per group) Data represent means±SEM.*p<0.05 *Ramp1^−/−^* (OVA) compared to *Ramp1^+/+^* (OVA), #p<0.05 *Calcrl^+/−^* (OVA) compared to *Calcrl^+/+^* (OVA).

As previously stated, others have found that a loss of CGRP resulted in an attenuation of airway resistance in a model of OVA-induced asthma [17]. Since CGRP signals via the *Ramp1*/*Calcrl* receptor interface, we hypothesized that a loss of *Ramp1* would lead to reduced airway resistance in the same model. Therefore, we sensitized wildtype and *Ramp1^−/−^* animals as described in the methods section. These sensitized animals then inhaled either aerosolized PBS or 1% OVA to stress the airways. When we assessed the airway resistance (R_AW_) in response to MCh inhalation, we found that *Ramp1^−/−^* animals challenged with an inhalation of 1% OVA had maximum R_AW_ measurements that were significantly lower than wildtype animals challenged with 1% OVA and similar to control animals that inhaled PBS (**Figure 3A**). These data support the findings of Aoki-Nagase et al [17] that a loss of CGRP signaling results in decreased airway resistance.

To further support our findings, we also tested whether a reduction in the G-protein coupled receptor that associates with *Ramp1* to form the CGRP receptor, *Calcrl*, also attenuated the R_AW_ in this model. Since *Calcrl^−/−^* animals are embryonic lethal during mid-gestation [10], we used *Calcrl^+/−^* mice, which express 50% less *Calcrl* than wildtype littermates [10], to investigate the importance of the receptor component in this phenomenon. When we measured the R_AW_ after sensitizing and challenging wildtype and *Calcrl^+/−^* mice as described previously, we found that the *Calcrl^+/−^* animals challenged with 1% OVA inhalation had reduced R_AW_ after inhaling 40 mg/mL of MCh compared to similarly challenged wildtype animals (**Figure 3B**), indicating that CGRP and both receptor components, *Calcrl* and *Ramp1*, are important contributors to airway hyperresponsiveness in wildtype animals.

One contributing factor to airway hyperresponsiveness is the contraction of smooth muscle cells in the small airways. Therefore, we were interested in determining whether CGRP acts on *Calcrl* expressed by the smooth muscle cells to drive the hyperresponsiveness of the airways. To address this question, we crossed mice with two floxed *Calcrl* alleles [11] to a smooth muscle-specific Cre line, the SM22-Cre line [19], resulting in mice with a smooth muscle-specific loss of *Calcrl*, and therefore, a loss of CGRP signaling in this cell type. In contrast to our results with the global *Calcrl^+/−^* mice, *SM22-Cre:Calcrl^fl/f^* mice challenged with 1% OVA experienced dramatically increased R_AW_ with MCh challenge in a manner similar to wildtype animals challenged with 1% OVA (**Figure 3C**). These data indicate that CGRP is not acting directly on the smooth muscle cells to drive airway constriction.

### Loss of *Ramp1* diminished lung inflammation

**Figure 4:**
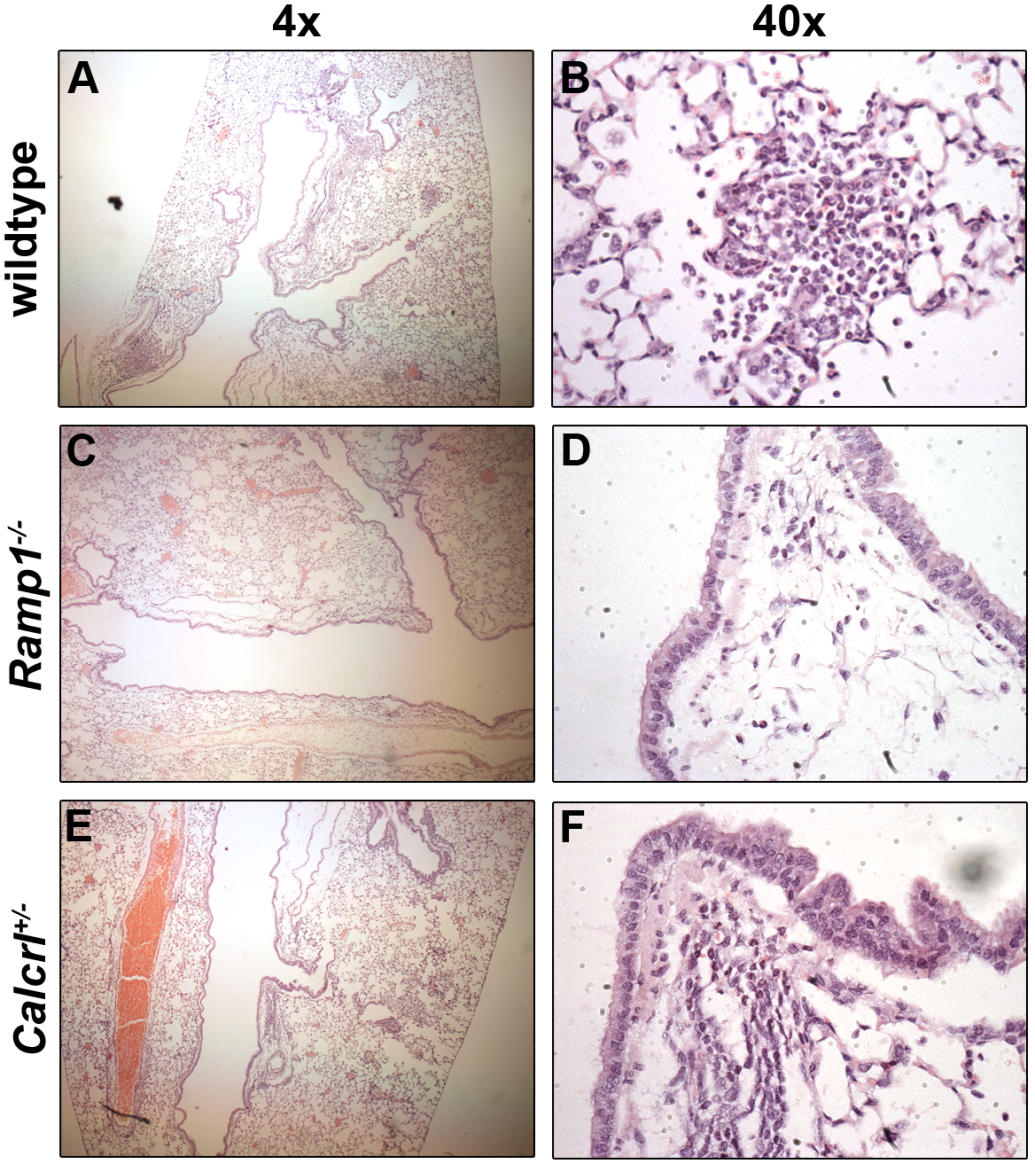
Lung histology of wildtype, *Ramp1^−/−^*, and *Calcrl^+/−^* animals after sensitization and methacholine challenge. Wildtype (**A** and **B**), *Ramp1^−/−^* (**C** and **D**)*­*, and *Calcrl^+/−^* (**E** and **F**) mice were sensitized with ovalbumin prior to a five day challenge period during which mice inhaled aerosolized ovalbumin as described in the methods. After measuring airway resistance in response to a methacholine challenge, lungs were harvested for histological analysis. Low power images obtained at 4x magnification are shown in panels **A**, **C**, and **E**, while high power images taken at 40x magnification are shown in panels **B**, **D**, and **F**.

**Figure 5:**
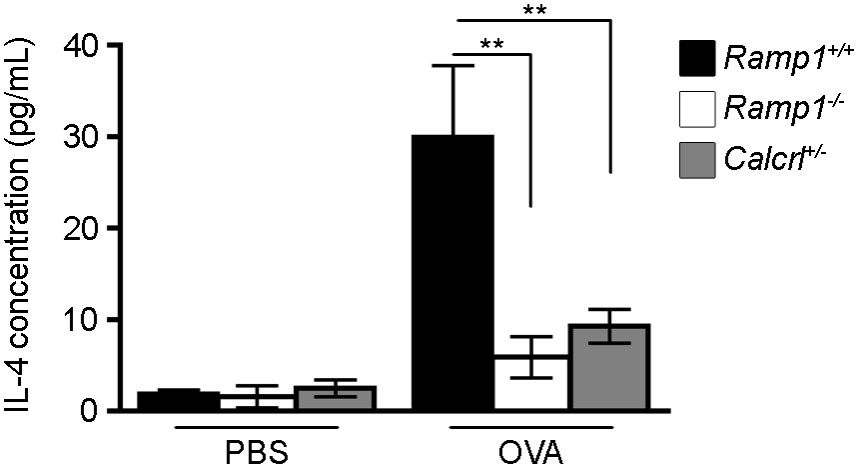
Reduced IL-4 levels from bronchoalveolar lavage fluid of *Ramp1^−/−^* and *Calcrl^+/−^* mice compared to wildtype. After sensitizing wildtype, *Ramp1^−/−^*, and *Calcrl^+/−^* mice to ovalbumin and challenging them with either saline (PBS) or ovalbumin as described in the methods section, the airway resistance was determined prior to collection of bronchoalveolar lavage (BAL) fluid. Using an ELISA assay, the concentration of IL-4 in the BAL fluid was determined for each group in each condition. (n = 4 per group) Data represent means±SEM. **p<0.01.

Since the OVA-induced model of allergic asthma results in the infiltration of inflammatory cells and the production of many cytokines, including IL-4 [27], we were interested in determining whether the decreased R_AW_ of the *Ramp1^−/−^* and *Calcrl^+/−^*mice was associated with a reduction in inflammatory cells and cytokines. We began by assessing the degree of inflammatory cell infiltration by examining H&E stained lung sections (**Figure 4**). While examining low power fields of wildtype (**Figure 4A**), *Ramp1^−/−^* (**Figure 4C**), and *Calcrl^+/−^* (**Figure 4E**) lungs, we noticed patches of infiltrating cells present throughout the lobe of the wildtype mice, while these patches were absent in *Ramp1^−/−^* and *Calcrl^+/−^* mice. Upon closer examination (**Figure 4B, D, and F**), it is clear that wildtype animals have patches of inflammatory cells present in the airways, while these inflammatory cells appear to be absent in the lungs of *Ramp1^−/−^* and *Calcrl^+/−^* animals. Finally, since IL-4 is a potent cytokine for T_H_2 cells, we assessed the levels of IL-4 in the BAL fluid. Using an ELISA, we found that compared to PBS-challenged mice, IL-4 was increased by approximately 15-fold in wildtype mice challenged with OVA (**Figure 5**). Interestingly, the concentration of IL-4 in the BAL fluid of the OVA-challenged *Ramp1^−/−^* and *Calcrl^+/−^* mice was elevated only 3- to 4- fold compared to PBS-challenged animals (**Figure 5**). Collectively, these data support the conclusion that a loss of CGRP signaling via the *Ramp1*/*Calcrl* interface results in a reduction of inflammation, thereby contributing to the reduced R_AW_ after MCh challenge.

### Immune cells in the lung express RAMP1 and CLR

**Figure 6:**
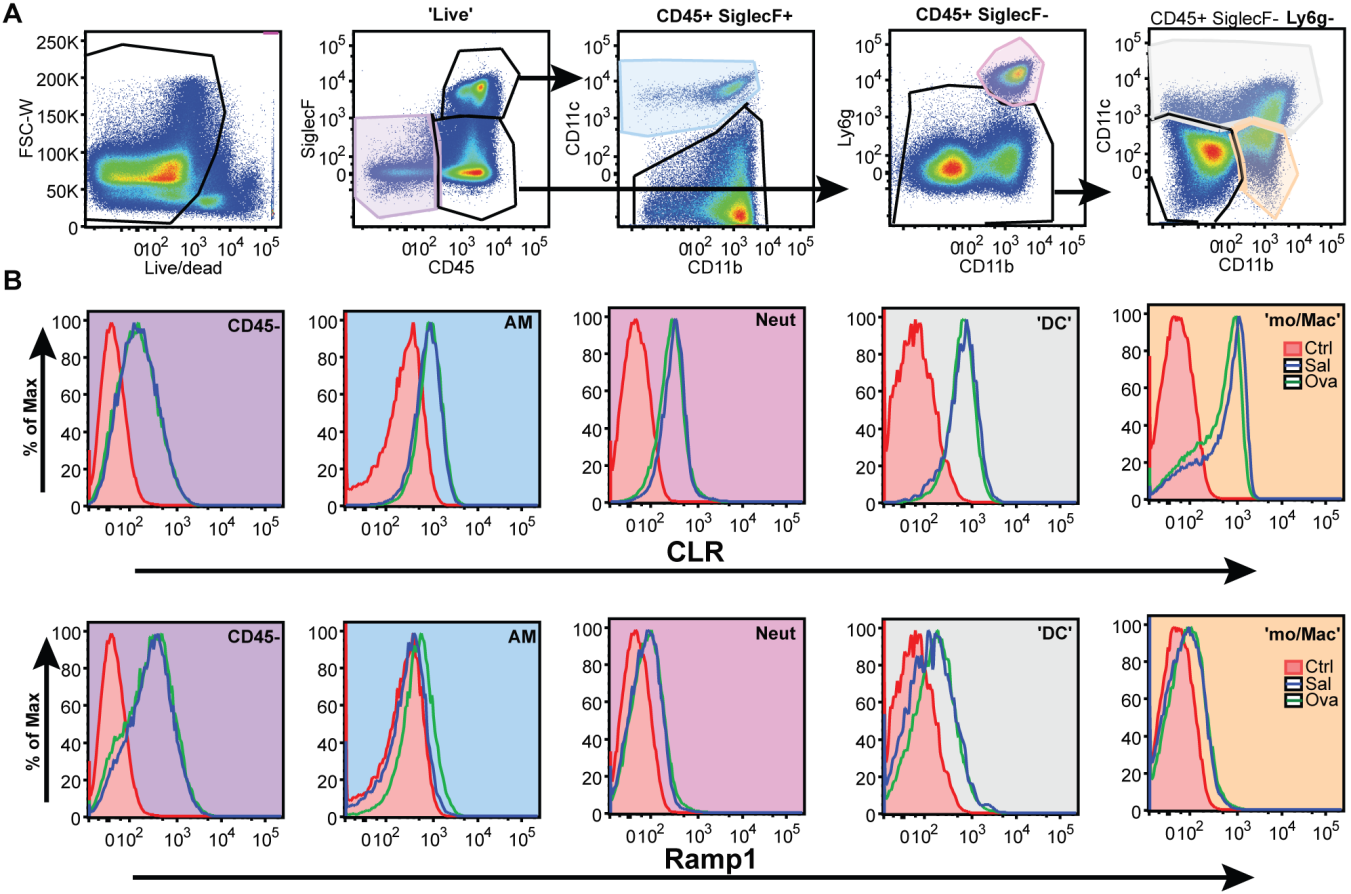
Expression of RAMP1 and CLR in lung tissue. **A.** Flow cytometry gating strategy to determine cell populations within the lung. Arrows highlight the parent population being defined. **B.** Representative histograms showing the surface expression of CLR and RAMP1 in saline (blue line; n = 2) and OVA challenged (green line; n = 3) mice. The control in tinted red is a secondary only antibody stain. The fill of the histogram box correlates with the colored population in the flow cytometry plots in A. AM = alveolar macrophage, Neut = neutrophil, ‘DC’ = dendritic cell, ‘mo/Mac’ = monocytes and monocyte-derived macrophage.

We were interested in determining whether CLR and RAMP1 were expressed in endothelial or inflammatory cells given the fact that the reduction or loss of these proteins reduced inflammation and airway constriction and that this effect did not seem dependent on the expression of CLR on smooth muscle cells. Through a panel of antibody stains focused on immune cell populations in the lung, we were able to study expression of RAMP1 and CLR on inflammatory cells. Using the gating strategy shown (**Figure 6A**), we were able to identify epithelial, endothelial, and inflammatory cells. CD45- cells, which include epithelium and endothelium, are positive for both CLR and RAMP1 (**Figure 6B**). Interestingly, alveolar macrophages, neutrophils, dendritic cells, and monocytes also expressed CLR, and to a lesser extent, RAMP1 (**Figure 6B**). We also noted that RAMP1 and CLR expression did not change following allergen challenge with ovalbumin (**Figure 6B**). Together, these data indicate that CLR and RAMP1 are expressed by endothelial, and to some extent inflammatory cells, indicating that CGRP signaling in these cell populations may be responsible for driving the observed airway hyperresponsiveness.

## Discussion

In this study, we have demonstrated that the loss of RAMP1 in sensitized animals results in a reduction of the airway resistance compared to sensitized wildtype controls, indicating that RAMP1-mediated signaling plays an important role in the hyperresponsiveness of the airways in this animal model of allergic asthma. Additionally, we found evidence that the loss of RAMP1 and reduced CLR attenuated lung inflammation. Importantly, endothelial and immune cells of the lung express RAMP1 and CLR before and after ovalbumin challenge. Together, these data uncover a pathophysiologically relevant function for RAMP1 and CLR in airway resistance.

RAMP1 is known to interact with CLR to form a receptor for CGRP, indicating that aberrant CGRP signaling may contribute to the pathology of asthma. Indeed, several studies have investigated the role of CGRP in the context of allergic asthma. In one such study, Aoki-Nagase et al demonstrated that the loss of α-CGRP in mice resulted in an amelioration of airway hyperresponsiveness following airway sensitization [17]. Although Aoki-Nagase et al did not find difference in inflammation [17] as we did, this can perhaps be explained by the fact that RAMPs can interact with other GPCRs [28], and therefore the RAMP1-mediated effects on inflammation could be driven by a different receptor that has yet to be identified. Furthermore, Bonner et al provide additional support for the theory that increased CGRP signaling contributes to the pathology of allergic asthma in a study of RAMP1 expression and localization in human asthma patients [24]. Consistent with our results, this study revealed that human airway epithelial cells express RAMP1 [24], and are therefore, able to mediate responses to CGRP. Interestingly, the authors found that in response to CGRP treatment, RAMP1 surface expression was reduced due to receptor internalization [24]. Additionally, this study revealed that human asthma patients displayed less RAMP1 on the surface of airway epithelial cells in response to trigger peptide inhalation compared to non-asthmatic controls [24], indicating that enhanced CGRP signaling via RAMP1-mediated signaling contributes to asthma pathology. Furthermore, Kay AB et al [29] found that human patients, who responded to allergen exposure, in this case cat allergens, had increased expression of CGRP. Interestingly, Kay AB et al found that CGRP expression was primarily increased in T cells, as well as the epithelium [29], further supporting the importance of these cell populations in modulating the effect of CGRP in allergic asthma. Finally, recent work performed by Bonner K et al [30] has revealed that CCL17 can induce CGRP expression in lung epithelial cells via CCR4, identifying one mechanism of CGRP regulation. Together, these recent studies indicate that CGRP signaling, presumably via inflammatory and lung epithelial cells, plays an important role in the pathogenesis of allergic asthma, further supporting our work presented here.

In our current study, we demonstrated that heterozygosity of CLR resulted in reduced airway resistance following sensitization and challenge. It is well known that, in addition to interacting with RAMP1 to form a receptor for CGRP, CLR can interact with RAMP2 and RAMP3 to form receptors for AM. Therefore, it is possible that some of the reduced airway resistance observed in the CLR heterozygotes is due to a reduction in AM signaling. Several years ago, Yamamoto et al published a study investigating the effect of AM heterozygosity on allergic asthma [31]. This study revealed that a 50% reduction in AM signaling resulted in an exacerbation of airway resistance [31], indicating that AM signaling may be a protective peptide against asthma. One possible explanation for the discrepancy between our data on CLR heterozygotes and the data regarding AM heterozygotes by Yamamoto et al is that the negative effects of CGRP signaling on asthma might be more potent than the protective effects of AM, and therefore diminished CGRP signaling may provide a greater benefit to airway resistance than the corresponding reduction in protective AM signaling. Additionally, it is possible that the expression of RAMP2 and RAMP3 is less robust in the endothelial cells of the lung compared to RAMP1; resulting in a preference of CGRP signaling over AM signaling and therefore biasing the phenotype towards that of the *Ramp1^−/−^* animals rather than towards the phenotype of the *Adm^+/−^* animals.

Pro- and anti-inflammatory signals play a critical role in dictating whether and to what extent allergic inflammation will occur. IL-4 is a potent pro-inflammatory cytokine that is responsible for activating T_H_2 cells [32], inducing mucin gene expression [33], enhancing the secretion of IgE [34], as well as up-regulating IgE receptor expression [35]. Together, these effects promote an immune response in the lungs and contribute to airway hyperresponsiveness in asthma. In our study, we found that *Ramp1^−/−^* animals had significantly less IL-4 present in the BAL, indicating that the loss of RAMP1 signaling attenuates the inflammatory response to inhaled triggers, which, in turn, prevents airway hyperreponsiveness. Our finding that inflammatory cells within the lung express RAMP1 and CLR, suggesting that direct action of CGRP on inflammatory cells promotes an inflammatory response within the lung, further supports this.

In summary, we have shown that a loss of RAMP1 signaling in a model of allergic asthma results in improvements in both airway resistance and inflammation after challenge. Additionally, a reduction of CLR in the lung also reduced the airway resistance, indicating that the CLR/RAMP1 complex, which is the receptor for CGRP, is a major mediator of pathological signals in allergic asthma. Finally, we report that endothelial as well as inflammatory cells within the lung express both RAMP1 and CLR, indicating that action of CGRP on these cell populations may contribute to the observed pathology in this model of allergic asthma. Since drug compounds can be developed to specifically target RAMP-receptor interfaces, compounds targeting the CLR/RAMP1 interface may be useful in providing relief from asthma.
